# Increased WIP1 Expression With Aging Suppresses the Capacity of Oocytes to Respond to and Repair DNA Damage

**DOI:** 10.3389/fcell.2021.810928

**Published:** 2021-12-24

**Authors:** Jiyeon Leem, Guang-Yu Bai, Jae-Sung Kim, Jeong Su Oh

**Affiliations:** ^1^ Department of Integrative Biotechnology, Sungkyunkwan University, Suwon, South Korea; ^2^ Biomedical Institute for Convergence at SKKU (BICS), Sungkyunkwan University, Suwon, South Korea; ^3^ Division of Radiation Biomedical Research, Korea Institute of Radiological and Medical Sciences, Seoul, South Korea

**Keywords:** oocyte, DNA repair, WIP1, aging, oocyte quality

## Abstract

If fertilization does not occur for a prolonged time after ovulation, oocytes undergo a time-dependent deterioration in quality *in vivo* and *in vitro*, referred to as postovulatory aging. The DNA damage response is thought to decline with aging, but little is known about how mammalian oocytes respond to the DNA damage during *in vitro* postovulatory aging. Here we show that increased WIP1 during *in vitro* postovulatory aging suppresses the capacity of oocytes to respond to and repair DNA damage. During *in vitro* aging, oocytes progressively lost their capacity to respond to DNA double-strand breaks, which corresponded with an increase in WIP1 expression. Increased WIP1 impaired the amplification of *γ*-H2AX signaling, which reduced the DNA repair capacity. WIP1 inhibition restored the DNA repair capacity, which prevented deterioration in oocyte quality and improved the fertilization and developmental competence of aged oocytes. Importantly, WIP1 was also found to be high in maternally aged oocytes, and WIP1 inhibition enhanced the DNA repair capacity of maternally aged oocytes. Therefore, our results demonstrate that increased WIP1 is responsible for the age-related decline in DNA repair capacity in oocytes, and WIP1 inhibition could restore DNA repair capacity in aged oocytes.

## Introduction

DNA double-strand breaks (DSBs) are often generated in genomic DNA during DNA replication or transcription or upon exposure to genotoxic agents such as ionizing radiation or chemicals. Because unrepaired DSBs can cause cell death, mutations, and genomic instability that can contribute to cancer, cells immediately recognize DSBs and recruit various proteins at the damaged chromatin regions to engage in the DNA repair process. This signaling cascade is initiated by the MRN (MRE11-RAD50-NBS1) complex, which senses and binds to DSB sites. The MRN complex recruits and activates ATM kinase, which then phosphorylates Ser-139 of histone H2AX (*γ*-H2AX) in the chromatin surrounding the DSB site. The phosphorylated tail of *γ*-H2AX creates a docking platform for the mediator protein MDC1, which recruits additional ATM and MRN complexes to the vicinity of the DSB, thereby amplifying ATM recruitment and activation and spreading *γ*-H2AX to adjacent chromatin (Stucki et al., 2005; Polo and Jackson, 2011; Panier and Boulton, 2014). This stepwise amplification of *γ*-H2AX signaling ensures the repair of damaged DNA by stabilizing the broken ends and recruiting repair factors (Celeste et al., 2003).

Once the DNA damage is repaired, *γ*-H2AX signaling is no longer required and must be reversed to return the cell to a normal state. This is mainly achieved by the type 2C protein phosphatase WIP1 (Lu et al., 2008). WIP1 dephosphorylates multiple proteins, including *γ*-H2AX and ATM, which terminates the DNA damage response (Lu et al., 2008; Macurek et al., 2010; Moon et al., 2010). Therefore, WIP1 is a major homeostatic regulator of the DNA damage response.

It is well documented that following ovulation, oocytes at prophase I resumes meiosis, undergoing a series of events that involves germinal vesicle (GV) breakdown (GVBD) and first polar body extrusion (PBE) to reach metaphase of the second meiosis (MII) stage. The ovulated MII oocytes await fertilization by the sperm either in the oviduct of the female reproductive tract or *in vitro* culture media during assisted reproductive technology (ART) procedure. If no fertilization occurs at an appropriate time, oocytes progressively undergo a time-dependent deterioration in quality, referred to as postovulatory aging (Lord and Aitken, 2013). Because DNA damage in oocytes can cause serious embryonic abnormalities that lead to birth defects, abortion, or infertility (Tease, 1983; Jacquet et al., 2005), the ability of oocytes to detect and repair DNA damage is essential in preserving reproductive capacity and the genetic fidelity of embryos. However, little is known about DNA damage response and repair in oocytes during *in vitro* postovulatory aging. In this work, we found that both *in vitro* postovulatory aging and maternal aging increase WIP1 expression in oocytes, which suppresses the capacity of the oocytes to respond to and repair DNA damage. We also show that WIP1 inhibition can enhance the DNA repair capacity of aged oocytes.

## Materials and Methods

### Oocyte Collection and Culture

All procedures for mouse care and use were approved by and conducted in accordance with guidelines from the Institutional Animal Care and Use Committees of Sungkyunkwan University (approval ID: SKKUIACUC 2019-11-07-1 and SKKUIACUC 2020-01-23-1). To collect fresh MII oocytes, 3- to 4-week old CD1 female mice (Koatech, Korea) were superovulated using an injection of 5 IU of pregnant mare’s serum gonadotropin (PMSG) followed by an injection of 5 IU of human chorionic gonadotropin 46–48 h later. After another 14–15 h, oocyte-cumulus complexes were collected from the oviducts, and the cumulus cells were removed using hyaluronidase. For *in vitro* aging, fresh MII oocytes were cultured in M16 medium covered with mineral oil at 37°C in a 5% CO_2_ atmosphere for up to 24 h. To obtain young and maternally aged oocytes, 8-week-old and 12-month-old CD1 mice were used, respectively.

For GV stage oocytes, immature oocytes were collected from the follicles 46–48 h after PMSG injection and recovered in M2 medium supplemented with 100 µM of 3-isobuthly-1-methylxanthine (IBMX). For *in vitro* maturation, GV oocytes were cultured in IBMX-free M2 medium covered with mineral oil at 37°C in a 5% CO_2_ atmosphere for 16 h.

To induce DNA damage, MII oocytes were exposed to 50 μg/ml etoposide for 15 min. For recovery, the etoposide-treated oocytes were washed and cultured in fresh M2 medium for 1 h with or without 5 µM GSK2830371 (Tocris). When needed, 10 µM ATM inhibitor (KU55933, Selleckchem), 50 µM NHEJ inhibitor (NU7441, Selleckchem) or 50 µM HR inhibitor (B02, Tocris) was added to the MII oocytes with GSK2830371 during recovery. Control oocytes were treated with dimethyl sulfoxide (DMSO). All chemicals and reagents were purchased from Sigma-Aldrich unless otherwise stated.

### mRNA Preparation and Microinjection

The full-length cDNA encoding human WIP1 was purchased from Korea Human Gene Bank and subcloned into the pRN3-mCherry vector. The mRNA was prepared from the T3 promoter of the vector using the mMESSAGE mMACHINE™ T3 Transcription kit (Thermo Fisher Scientific) and purified using an RNA purification kit (MACHEREY-NAGEL). GV oocytes were injected with mRNA for mCherry or WIP1-mCherry using a FemtoJet microinjector (Eppendorf) and hydraulic micromanipulator (Narishige) mounted onto a Leica inverted microscope (DMIRB). We injected 5–10 pL of mRNA (∼800 ng/μl) into the cytoplasm of the oocytes.

### Immunofluorescence Analysis

Oocytes were fixed with 4% paraformaldehyde in phosphate-buffered saline (PBS) for 10 min and permeabilized with 0.1% Triton X-100 and 0.01% Tween-20 for 20 min at room temperature. They were then blocked with 3% bovine serum albumin (BSA)-supplemented PBS for 1 h and incubated with anti-acetylated *a*-tubulin (1:1,000), anti-*γ*-H2AX (1:250, Abcam), anti-p-ATM (1:100, Abcam), or anti-MDC1 (1:250, Abcam) antibodies overnight at 4°C. After they were washed three times, the oocytes were incubated with Alexa Fluor–conjugated 488 secondary antibodies (1:500, Jackson ImmunoResearch) at room temperature for 2 h. Finally, the oocytes were counterstained with DAPI, mounted on glass slides, and observed under an LSM 700 laser scanning confocal microscope (Zeiss) with a C-Apochromat 63×/1.2 water immersion objective. To measure the fluorescence intensity, images were captured with the same laser power, and the mean intensity of the fluorescence signals was measured. Data were analyzed using ZEN 2012 Blue (Zeiss) and ImageJ software (National Institutes of Health) under the same processing parameters.

For mitochondrial staining, oocytes were incubated with Mitochondrial Staining Reagent-Red-Cytopainter (Abcam) for 1 h at 37°C and observed under confocal microscopy.

### Immunoblotting Analysis

For immunoblotting, 50 oocytes per sample were collected. The oocytes were lysed in 5 μl of 2 x reducing SDS loading buffer and heated at 95°C for 10 min. Proteins were separated by 8% SDS-PAGE and then transferred onto polyvinylidene difluoride membranes (Immobilon). The blots were blocked in 3% BSA in tris-buffered saline with Tween-20 (TBST) for 1 h at room temperature and incubated with anti-β-actin (1:5,000, Cell Signaling) or anti-WIP1 (1:1,000, Abcam) antibodies overnight at 4°C. After being washed three times in TBST, the blots were incubated with HRP-conjugated secondary antibodies (1:5,000, Jackson ImmunoResearch) for 1 h at room temperature. Immunodetected proteins were visualized on films using ECL Plus (GE Healthcare). The band intensities were quantified using ImageJ and normalized to loading controls.

### TUNEL Assay

The TUNEL assay was performed using an *In Situ* Cell Death Detection kit (Roche) according to the manufacturer’s instructions. Briefly, MII oocytes were exposed to acidic Tyrode’s solution for 2 min to remove zona pellucida. After a brief recovery in M2 medium, the oocytes were fixed on a glass slide in 1% paraformaldehyde in distilled water (pH 9.2) containing 0.15% Triton X-100 and 3 mM dithiothreitol for chromosome spreading. After drying in a humid chamber, the paraformaldehyde-fixed oocytes were washed three times with PBS and permeabilized with 0.15% Triton X-100 and 0.1% sodium citrate for 1 h on ice. After washing, the oocytes were incubated with fluorescent-conjugated terminal deoxynucleotide transferase dUTP for 2 h at 37°C. After counterstaining with DAPI, the oocytes were mounted on glass slides and observed using an LSM 700 laser scanning confocal microscope.

### Comet Assay

The comet assay was performed using an Alkaline CometAssay kit (Trevigen) according to the manufacturer’s instructions. Briefly, oocytes were mixed with melted agarose, placed on comet slides, and subjected to electrophoresis. The comet signals were visualized by staining with SYBR green (Invitrogen), and images were captured with a confocal microscope.

### Apoptosis Detection

Apoptosis was detected with a FITC Annexin V Apoptosis Detection kit II (BD Pharmingen). Oocytes were stained with Annexin V-FITC and propidium iodide for 30 min in the dark. After being washed three times, the oocytes were mounted on glass slides and counterstained with DAPI for confocal imaging.

### Intracytoplasmic Sperm Injection

Using BDF1 (C57BL/6×DBA/2) mice (8–10 weeks old) instead of CD1 mice, ICSI was performed as described previously (Bai et al., 2020). Briefly, sperm prepared from the cauda epididymis of 8- to 12-week-old BDF1 males was injected into MII oocytes using a micromanipulator with a Piezo impact drive (Prime Tech). After 30 min of recovery at room temperature, the sperm-injected oocytes were washed several times and cultured in KSOM (Millipore) medium at 37°C in a 5% CO_2_ atmosphere.

### Statistical Analysis

All statistical analyses were performed with GraphPad Prism 5.0 (GraphPad Software). Data are representative of at least three independent experiments unless otherwise specified, and each experimental group included at least 15 oocytes. Differences between two groups were analyzed by the Student’s t-test, and comparisons of more than two groups were analyzed by one-way ANOVA with Tukey’s post-hoc test. *p* < 0.05 was considered to be statistically significant.

## Results

### Oocytes Lose Their Capacity to Respond to DNA Damage, With a Corresponding Increase in WIP1, During *in vitro* Postovulatory Aging

**FIGURE 1:**
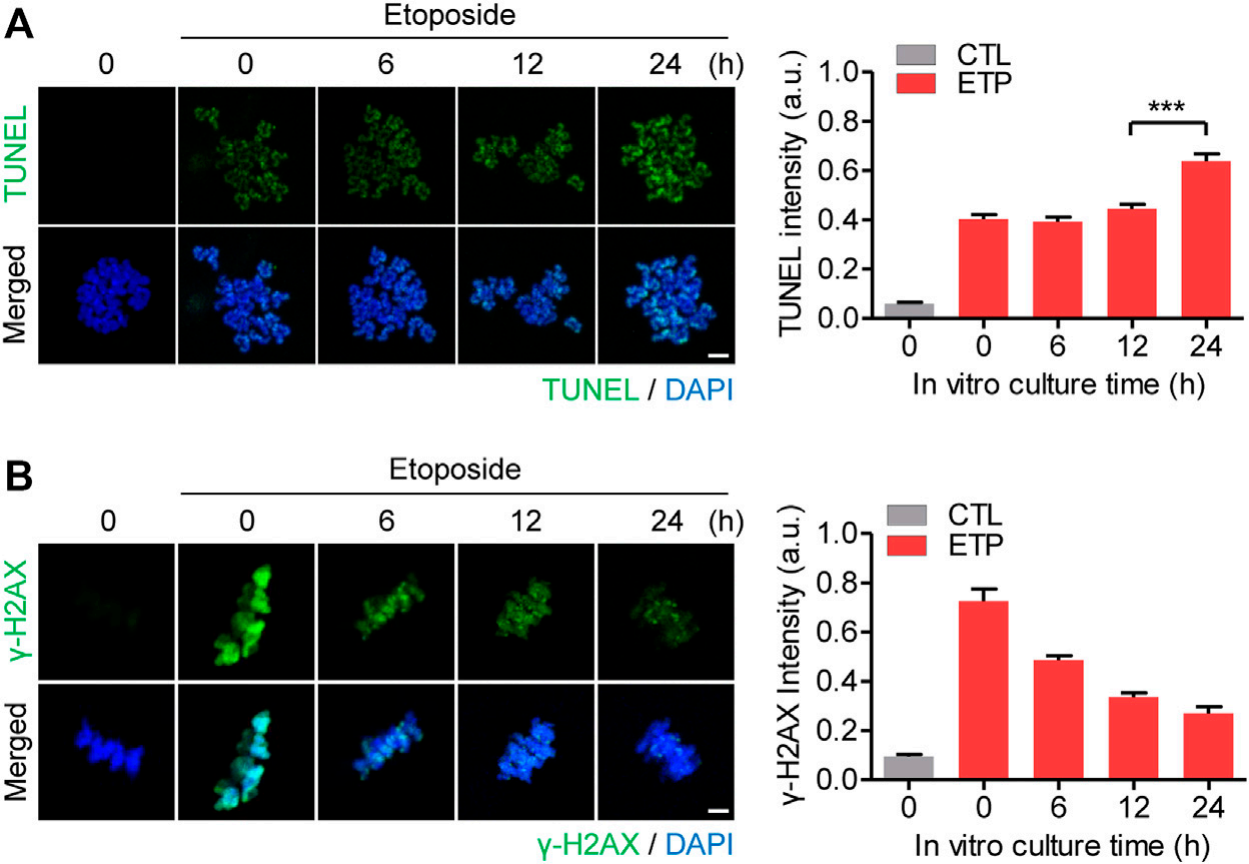
Oocytes lose their capacity to respond to DNA DSBs with *in vitro* aging. **(A,B)** MII oocytes were aged *in vitro* for 0, 6, 12, and 24 h, exposed to 50 μg/ml etoposide (ETP) for 15 min, and then subjected to either the TUNEL assay **(A)** or *γ*-H2AX staining **(B)**. Oocytes were treated with DMSO as a control (CTL). The intensity of the fluorescence signals was quantified, and representative images are shown. DNA was counterstained with DAPI. Scale bar, 10 μm. Data were analyzed by one-way ANOVA followed by Tukey’s post hoc test and are expressed as the mean ± SEM of three independent experiments. ***p < 0.0001.

Although an effective response to DNA damage is essential to maintain genomic integrity in all cells, including oocytes, their capacity to respond to DNA damage is thought to decline with age. To investigate whether that is the case in oocytes during *in vitro* postovulatory aging, MII oocytes were cultured *in vitro* for 0, 6, 12, and 24 h and then treated with etoposide for 15 min to induce DNA DSBs. The oocytes were then assessed for DNA damage using the terminal deoxynucleotidyl transferase dUTP nick end labeling (TUNEL) assay and *γ*-H2AX staining. The TUNEL assay showed that the degree of DNA damage following etoposide treatment was comparable between fresh and 12 h old oocytes but increased after 24 h of culture (Figure 1A). This result suggests that prolonged culture makes oocytes vulnerable to DNA damage. Unlike the TUNEL signals, *γ*-H2AX levels after etoposide treatment decreased gradually with increased culture time (Figure 1B). Given that *γ*-H2AX signaling is one of the initial responses to DSBs, that result suggests that oocytes progressively lose their capacity to respond to DNA damage during postovulatory *in vitro* aging.

**FIGURE 2:**
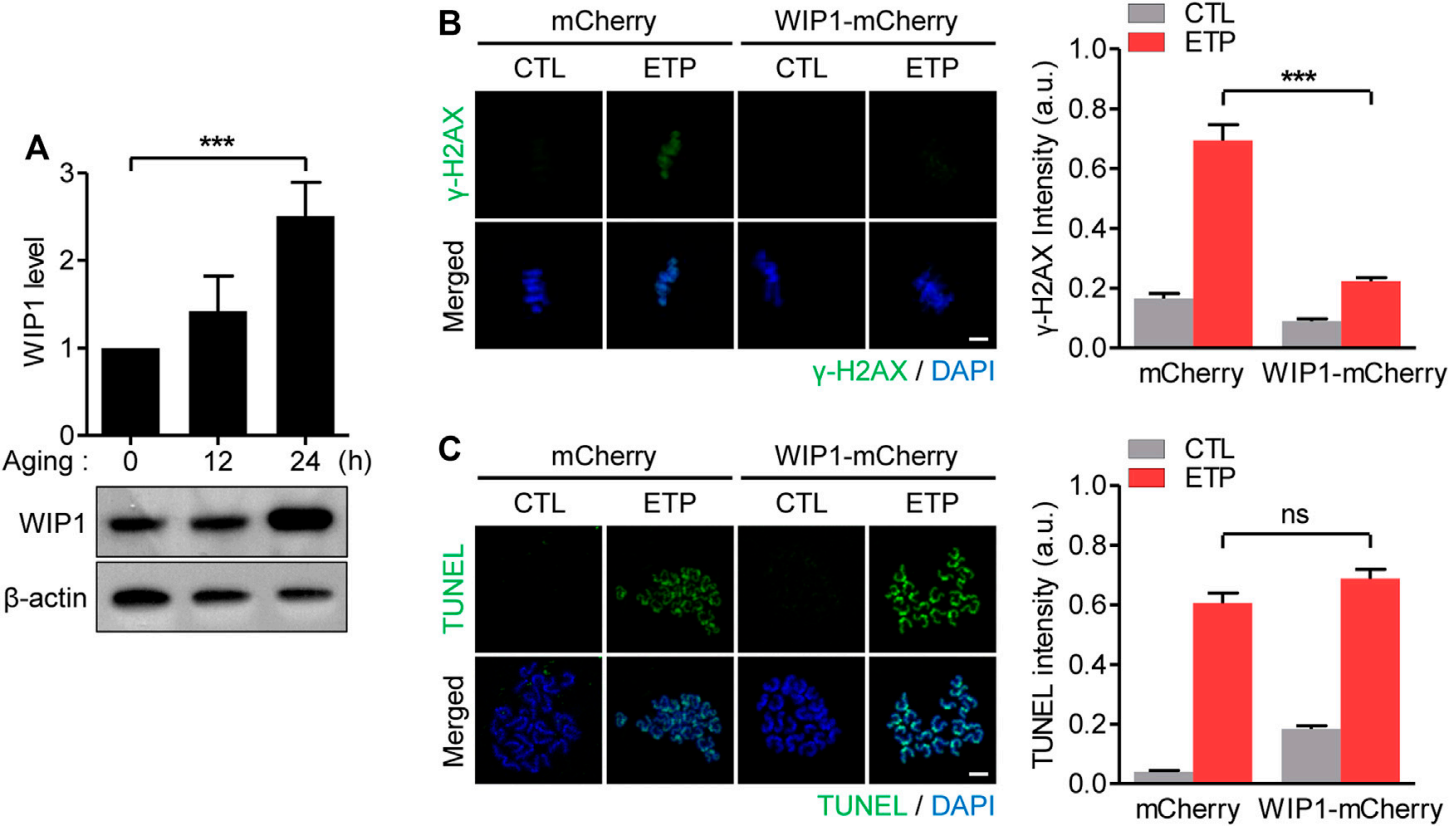
Decreased response to DNA damage in aged oocytes is associated with increased WIP1 levels. **(A)** Expression levels of WIP1 during *in vitro* aging for 0, 12 and 24 h were examined by immunoblot analysis using the WIP1 antibody, with *ß*-actin as a loading control. Each lane contains 50 oocytes. WIP1 levels normalized to *ß*-actin were quantified and expressed as the mean ± SEM from two independent experiments. **(B,C)** GV oocytes injected with mRNA encoding either WIP1-mCherry or mCherry were matured to the MII stage for 16 h to allow for protein overexpression. After 15 min of exposure to etoposide (ETP) or DMSO as a control (CTL), the *in vitro*–matured MII oocytes were subjected to *γ*-H2AX staining **(B)** or the TUNEL assay **(C)**. DNA was counterstained with DAPI. The fluorescence intensity normalized to the mean DAPI intensity was quantified and expressed as the means ± SEM from three independent experiments. Data were analyzed by one-way ANOVA followed by Tukey’s post hoc test. Scale bar, 10 μm ***p < 0.0001; ns, not significant.

Because WIP1 suppresses the DNA damage response in immature oocytes arrested in the G2/prophase (Leem et al., 2018), we wondered whether the reduced response to DNA damage in aged oocytes was associated with the WIP1 level. To investigate that possibility, we determined the WIP1 expression of oocytes during *in vitro* aging. Surprisingly, WIP1 levels were increased at 12 h of aging and markedly elevated after 24 h of aging (Figure 2A). To understand the correlation between the increased WIP1 level and the decreased DNA damage response, we ectopically overexpressed WIP1 and examined the DNA damage response in oocytes. Immature G2/prophase-arrested oocytes were injected with mRNA encoding either mCherry or WIP1-mCherry and matured to the MII stage to allow protein overexpression. Although the percentage of oocytes that matured to the MII stage decreased slightly after WIP1 overexpression, a significant portion of the oocytes overexpressing WIP1 progressed to the MII stage with the normal configuration of spindle and chromosomes (Supplementary Figure S1). Therefore, MII oocytes overexpressing WIP1 were used to investigate the effect of increased WIP1 levels on the DNA damage response. In the absence of etoposide treatment, *γ*-H2AX levels were barely detectable in both the control and WIP1-overexpressing oocytes. After etoposide treatment, *γ*-H2AX levels increased dramatically in the control oocytes, but the increase in *γ*-H2AX levels was diminished in the WIP1-overexpressing oocytes (Figure 2B). The TUNEL assay revealed that the degree of DNA damage following etoposide treatment was similar between the control and WIP1-overexpressing oocytes (Figure 2C). These results, along with the reduced *γ*-H2AX levels in response to the same degree of DNA damage in aged oocytes, suggest that increased WIP1 levels during *in vitro* aging suppresses the capacity of oocytes to respond to DNA damage.

### Increased WIP1 Delays Amplification of *γ*-H2AX signaling in response to DNA damage in postovulatory aged oocytes

**FIGURE 3:**
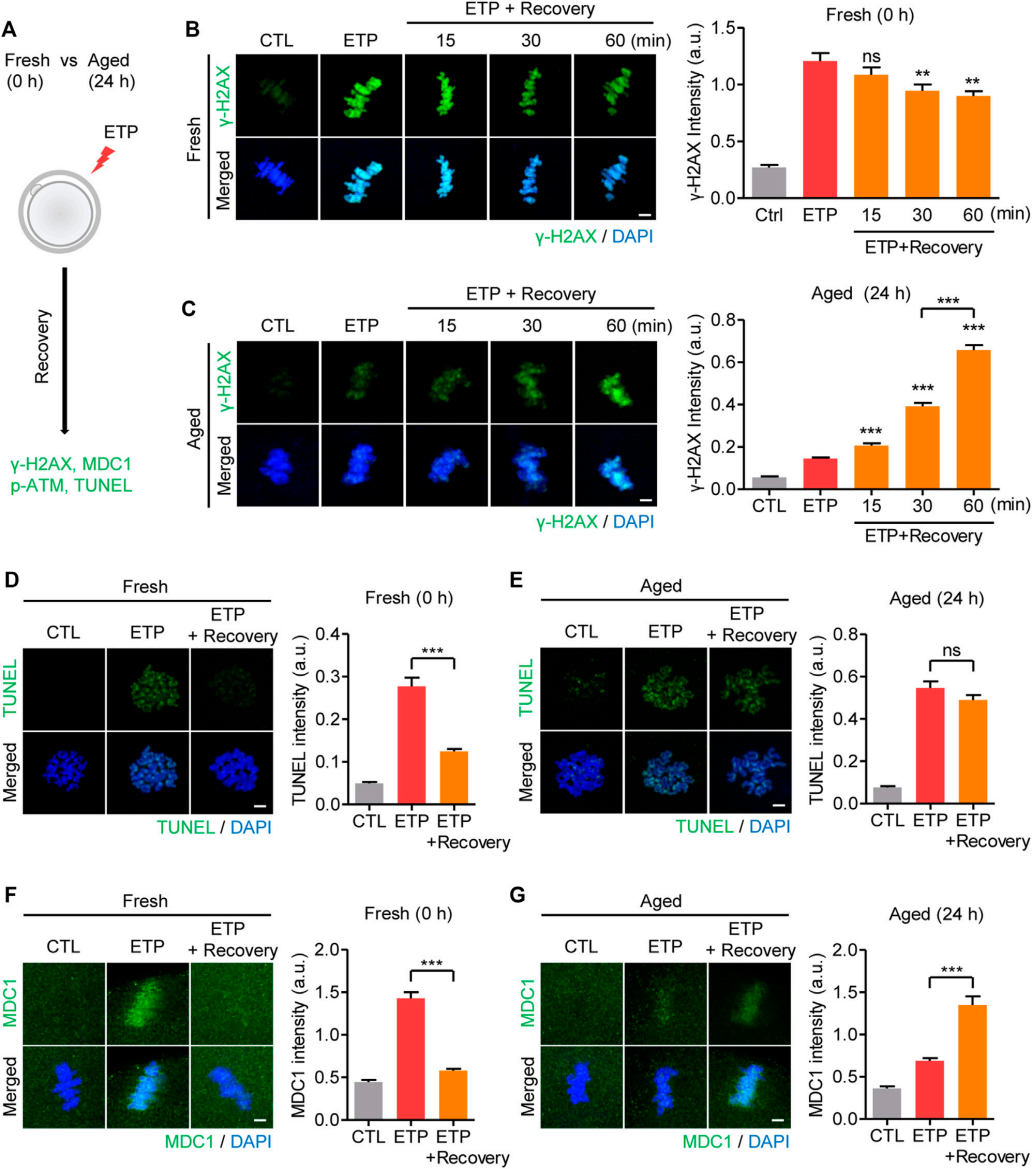
Increased WIP1 delays the amplification of *γ*-H2AX signaling in response to DSB in aged oocytes. **(A)** Scheme of the DSB recovery experiment. After 15 min of exposure to etoposide (ETP) or DMSO as a control (CTL), fresh oocytes and oocytes aged *in vitro* for 24 h were cultured in ETP-free medium for up to 1 h for recovery and then subjected to immunofluorescence staining or the TUNEL assay. **(B–G)** The fluorescence intensities of *γ*-H2AX **(B,C)**, TUNEL **(D,E)**, or MDC1 **(F,G)** signals were normalized to the mean DAPI intensity, and representative images from three independent experiments are shown. DNA was counterstained with DAPI. Data were analyzed by one-way ANOVA followed by Tukey’s post hoc test. **p < 0.001; ***p < 0.0001; ns, not significant. Scale bar, 10 μm.

To investigate how the decreased response to DSBs in aged oocytes affected DNA damage repair, MII oocytes were treated with etoposide and allowed to recover from the DNA damage (Figure 3A). In fresh oocytes, *γ*-H2AX levels increased immediately after etoposide treatment and decreased gradually as the cells recovered from the DNA damage (Figure 3B). However, the *in vitro*–aged oocytes exhibited a delayed increase in *γ*-H2AX levels during the recovery time (Figure 3C). To determine whether the increased *γ*-H2AX levels after recovery in aged oocytes were caused by a failure to repair the damaged DNA, we performed the TUNEL assay again. In fresh oocytes, TUNEL signals were significantly reduced after recovery, in agreement with the decrease in *γ*-H2AX levels during recovery (Figure 3D). However, in aged oocytes, despite the increasing *γ*-H2AX levels during recovery, the TUNEL signals did not change after recovery (Figure 3E). These results not only suggest that aged oocytes have a reduced ability to repair damaged DNA, but also imply that the *γ*-H2AX level did not represent the degree of DNA damage in aged oocytes. Given that the *γ*-H2AX signal generally spreads to adjacent areas of chromatin to amplify the DNA damage response, we wondered whether the delayed increase in *γ*-H2AX levels in aged oocytes was associated with the impaired amplification of the DNA damage response. To test that possibility, we compared the MDC1 levels immediately after 15 min exposure to etoposide with those after 1 h recovery from DNA damage, because MDC1 is a key player in the amplification of *γ*-H2AX signaling. In fresh oocytes, MDC1 levels increased rapidly after etoposide treatment and decreased after 1 h recovery (Figure 3F). In contrast, MDC1 was barely detectable immediately after etoposide treatment and increased after 1 h recovery in aged oocytes, consistent with delayed increase in *γ*-H2AX levels in aged oocytes (Figure 3G). In line with the MDC1 levels, p-ATM also exhibited delayed accumulation around chromosomes in aged oocytes (Supplementary Figure S2). Therefore, the diminished response to DNA damage in aged oocytes is associated with impaired amplification of the DNA damage response. To further investigate whether the delayed amplification of *γ*-H2AX signaling in aged oocytes is associated with increased WIP1 expression, we examined the MDC1 levels in WIP1-overexpressing oocytes. Similar to the results in aged oocytes, WIP1-overexpressing oocytes displayed a delayed increase in MDC1 levels after etoposide treatment (Supplementary Figure S3). Therefore, impaired amplification of *γ*-H2AX signaling is likely caused by the increased WIP1 levels in aged oocytes.

### Inhibition of WIP1 Enhances DNA Damage Repair in Postovulatory Aged Oocytes

**FIGURE 4:**
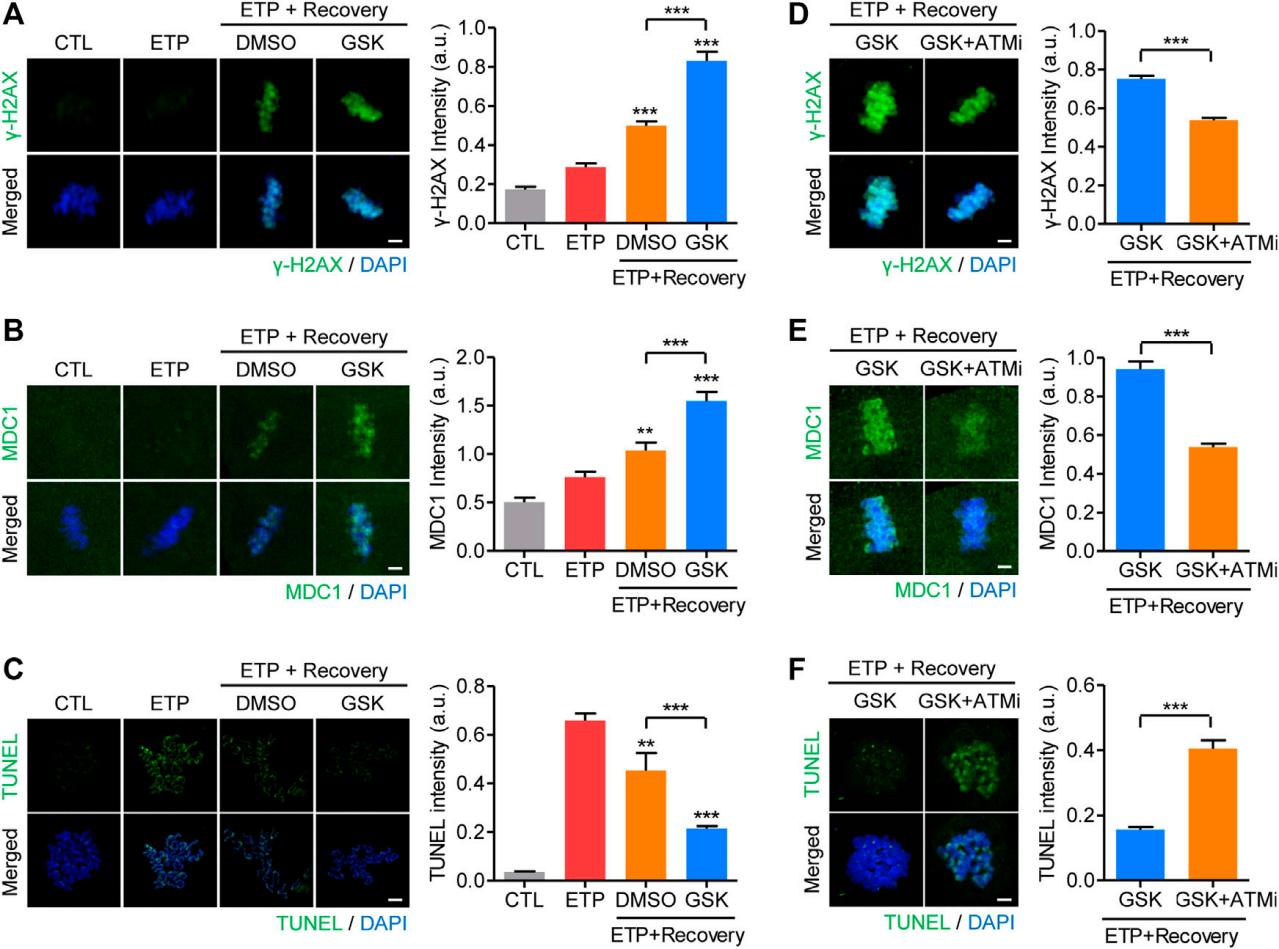
WIP1 inhibition promotes DNA damage repair in aged oocytes. **(A–C)** After treatment with etoposide (ETP) or DMSO as a control (CTL), oocytes aged *in vitro* for 24 h were cultured in ETP-free medium for 1 h with GSK2830371 (GSK) or DMSO. The intensities of *γ*-H2AX **(A)**, MDC1 **(B)**, and TUNEL **(C)** signals were normalized to the mean DAPI intensity, and representative images are shown. DNA was counterstained with DAPI. Scale bar, 10 μm **p < 0.001; ***p < 0.0001, calculated using one-way ANOVA with Tukey’s post hoc test. **(D–F)** Oocytes aged *in vitro* were co-treated with an ATM inhibitor (ATMi) and GSK2830371 (GSK) during recovery from etoposide (ETP). The intensities of *γ*-H2AX **(D)**, MDC1 **(E)** and TUNEL **(F)** signals were normalized to the mean DAPI intensity, and representative images are shown. DNA was counterstained with DAPI. Scale bar, 10 μm. Data are presented as the mean ± SEM of three independent experiments. ***p < 0.0001.

If the increase in WIP1 levels is the cause of an age-associated decline in DNA repair capacity in oocytes, we hypothesized that inhibiting WIP1 could improve the DNA repair capacity of aged oocytes. To investigate that possibility, MII oocytes aged for 24 h were treated with etoposide and allowed to recover from DNA damage with and without the WIP1 inhibitor GSK2830371. Unlike fresh oocytes, in which *γ*-H2AX signals were dramatically elevated in response to DNA damage (Figure 1), *γ*-H2AX levels in aged oocytes increased only marginally after etoposide treatment, consistent with an age-associated decline in the DNA damage response. However, the *γ*-H2AX levels did increase after 1 h of recovery, and that increase was more pronounced when WIP1 was inhibited during recovery (Figure 4A). Similar to *γ*-H2AX, MDC1 and p-ATM levels increased after 1 h of recovery, and that increase was more pronounced with WIP1 inhibition during recovery (Figure 4B and Supplementary Figure S2B). Importantly, in contrast to the *γ*-H2AX levels, the increased TUNEL signals found after etoposide treatment decreased after 1 h of recovery, and that decrease was more dramatic when WIP1 was inhibited during recovery (Figure 4C). A decrease in DNA damage after WIP1 inhibition was further confirmed by the comet assay (Supplementary Figure S4). Interestingly, ATM inhibition reversed all of the effects triggered by WIP1 inhibition during recovery (Figures 4D–F). Therefore, WIP1 inhibition could improve DNA damage repair in aged oocytes by promoting ATM-dependent amplification of *γ*-H2AX signaling.

To gain insights into the repair mechanisms of oocytes under WIP1 inhibition, we treated MII oocytes with inhibitors targeting either NHEJ (NU7441) or HR (B02) during recovery. Although no change in *γ*-H2AX levels was detectable in oocytes treated with GSK2830371 and B02, *γ*-H2AX levels decreased in oocytes treated with GSK2830371 and NU7441 (Supplementary Figure S5). Thus, the increased repair capacity of oocytes induced by WIP1 inhibition could be abolished by inhibiting the NHEJ pathway, implying that MII oocytes repair DNA DSBs via the NHEJ pathway.

To further validate our finding that increased WIP1 is associated with an age-associated decline in DNA repair in oocytes, we compared *γ*-H2AX levels and TUNEL signals in WIP1-overexpressing oocytes. In line with the results obtained from *in vitro*–aged oocytes, the increase in *γ*-H2AX levels in the WIP1-overexpressing oocytes was more pronounced in the presence of GSK2830371 during recovery (Supplementary Figure S6A). In contrast, the increase in the TUNEL signal after etoposide treatment decreased after 1 h of recovery, and that decrease was more dramatic in the WIP1-overexpressing oocytes when WIP1 was inhibited during recovery (Supplementary Figure S6B). Taken together, our results suggest that the impaired DNA damage repair found in aged oocytes is a consequence of increased WIP1 levels, and therefore, WIP1 inhibition could enhance the DNA repair capacity of aged oocytes.

### Inhibition of WIP1 During *in vitro* Postovulatory Aging Improves Fertilization and Subsequent Early Embryo Development

**FIGURE 5:**
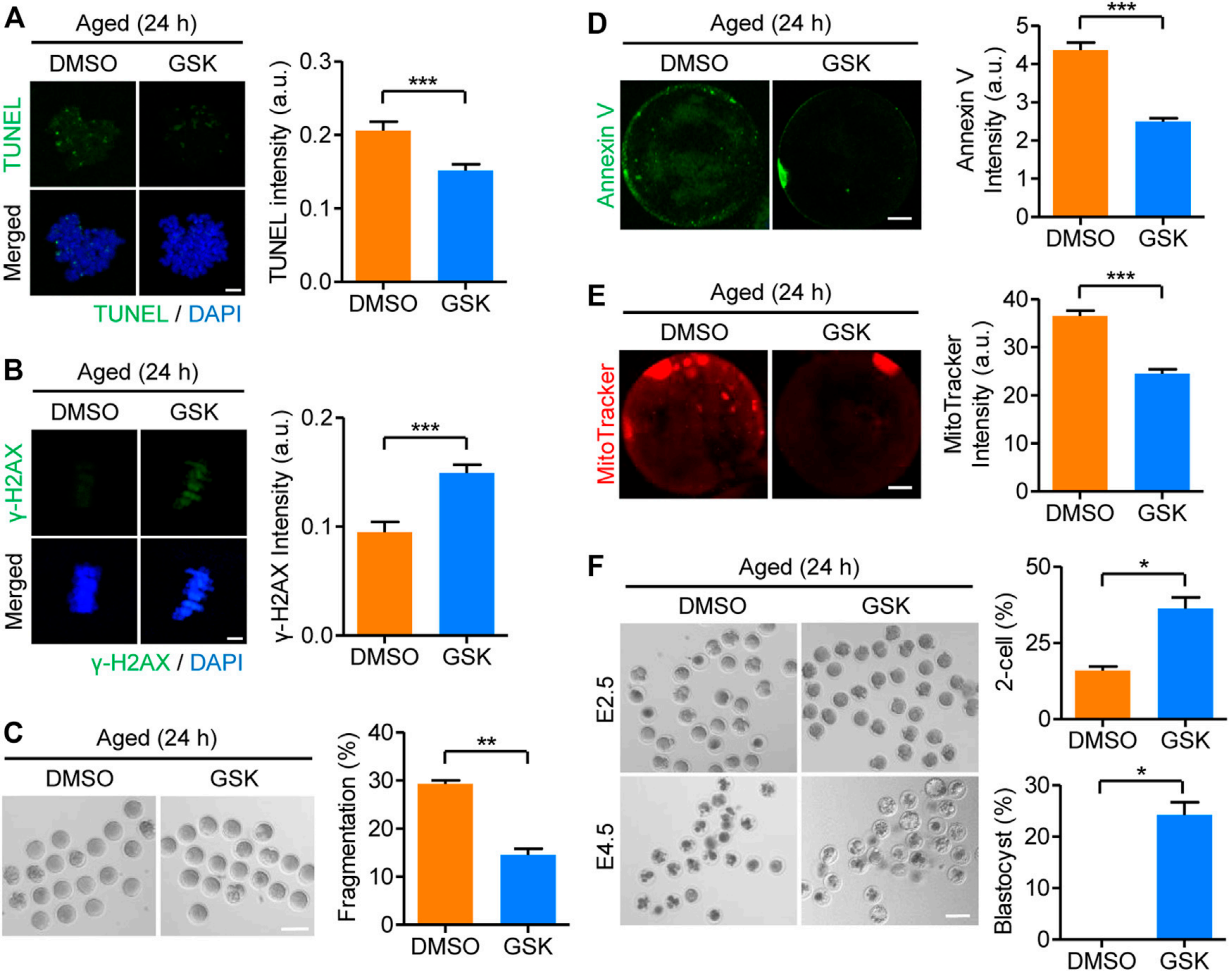
Prolonged inhibition of WIP1 during *in vitro* aging improves oocyte quality. MII oocytes were cultured *in vitro* for 24 h with GSK2830371 (GSK) or DMSO. **(A,B)** The intensities of TUNEL and *γ*-H2AX signals normalized to the mean DAPI intensity are shown using representative images. DNA was counterstained with DAPI. Scale bar, 10 μm. **(C)** Oocyte fragmentation was scored, and representative images are shown. Scale bar, 100 μm. **(D)** The intensity of Annexin V signals was scored, and representative images are shown. Scale bar, 20 μm. **(E)** The intensity of mitochondrial staining was scored, and representative images are shown. Scale bar, 20 μm. **(F)** The rate of 2-cell and blastocyst development is shown using representative images taken at 24 h (E2.5) and 72 h (E4.5) of culture after ICSI, respectively. Scale bar, 100 μm. Data are presented as the mean ± SEM from three independent experiments. *p < 0.05; **p < 0.001; ***p < 0.0001.

It is well established that DNA damage accumulates with aging due to oxidative stress over time. Therefore, we investigated whether WIP1 inhibition could prevent the accumulation of DNA damage during *in vitro* aging of oocytes. Consistent with the aging-associated accumulation of DNA damage, TUNEL signals, which were barely detectable in fresh oocytes, appeared in aged oocytes. However, the TUNEL signals significantly decreased when the oocytes were treated with GSK2830371 during *in vitro* aging (Figure 5A). In contrast to the TUNEL signals, *γ*-H2AX levels decreased in aged oocytes and increased after GSK2830371 treatment during *in vitro* aging (Figure 5B). Considering that reduced *γ*-H2AX levels in aged oocytes reflect low efficiency in DNA repair, these results suggest that prolonged inhibition of WIP1 during *in vitro* aging increases the DNA repair capacity of oocytes.

Having established that DNA damage induced by oxidative stress is one of the main causes of decline in oocyte quality during *in vitro* aging, our finding that WIP1 inhibition could increase the capacity of oocytes to repair DNA DSBs prompted us to investigate the effect of WIP1 inhibition on the quality of oocytes aged *in vitro*. To this end, MII oocytes were aged *in vitro* for 24 h in the presence of GSK2830371, and then oocyte quality was assessed by examining morphological abnormalities, chromosome and spindle abnormalities, the degree of apoptosis, and mitochondrial aggregation. Consistent with aging-associated quality decline, morphological abnormalities, including fragmentation, appeared in aged oocytes. However, the rate of oocytes with morphological abnormalities was decreased by WIP1 inhibition (Figure 5C). Similarly, the rate of apoptosis and mitochondrial aggregation decreased after WIP1 inhibition during *in vitro* aging (Figures 5D,E), as did abnormalities in chromosome and spindle organization (Supplementary Figure S7). To further investigate the effects of WIP1 inhibition on oocyte quality during *in vitro* aging, we performed intracytoplasmic sperm injection (ICSI) with the aged oocytes. Consistent with the improved oocyte quality observed with WIP1 inhibition, the rate of 2-cell and blastocyst development increased when oocytes were treated with GSK2830371 during *in vitro* aging (Figure 5F). Therefore, our results demonstrate that WIP1 inhibition during *in vitro* aging can prevent a decline in oocyte quality by increasing DNA repair capacity, thereby improving fertilization and embryo development.

### Inhibition of WIP1 Increases the DNA Repair Capacity of Oocytes From Aged Mice

**FIGURE 6:**
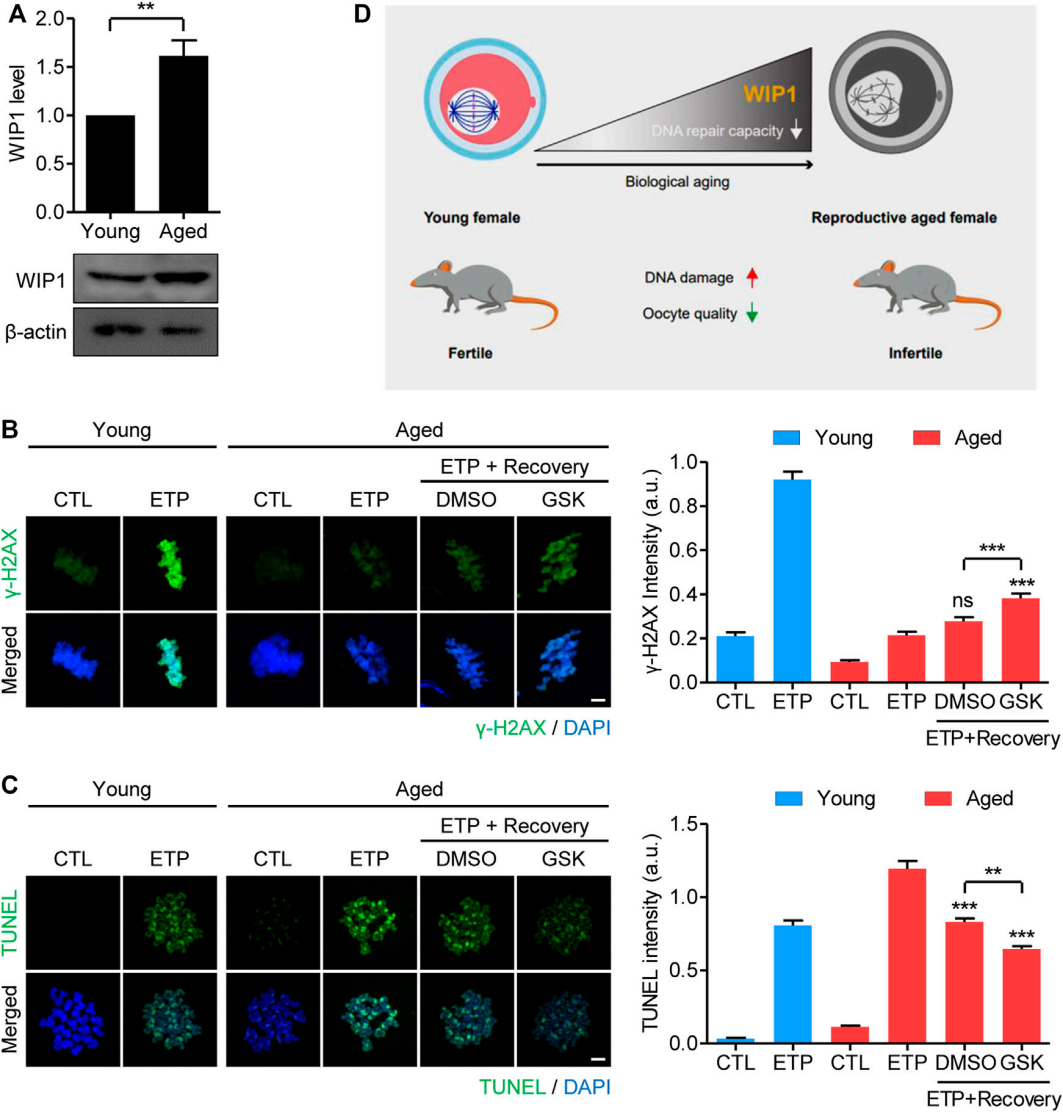
WIP1 inhibition increases the DNA repair capacity of oocytes from aged mice. **(A)** Increased WIP1 levels in maternally aged oocytes. MII oocytes were collected from young (8-week-old) and old (12-month-old) mice and subjected to immunoblot analysis using the WIP1 antibody, with *ß*-actin as a loading control. Each lane contains 50 oocytes. **(B,C)** Oocytes from young and old mice were treated with etoposide (ETP) or DMSO as a control (CTL) for 15 min. For recovery, the aged oocytes were cultured in ETP-free medium with GSK2830371 (GSK) or DMSO for 1 h after ETP exposure. The intensities of *γ*-H2AX and TUNEL, normalized to the mean DAPI intensity, are shown using representative images. DNA was counterstained with DAPI. Scale bar, 10 μm. Data were analyzed by one-way ANOVA followed by Tukey’s post hoc test and are expressed as the mean ± SEM of two independent experiments. **p < 0.001; ***p < 0.0001; ns, not significant. **(D)** A diagram showing the relationship between the aging-associated increase in WIP1 levels and the decline in DNA repair capacity in aged oocytes.

It is well known that postovulatory aging of oocytes has a similar phenotype of reproductive failure as oocyte aging caused by maternal aging (Miao et al., 2009; Lord and Aitken, 2013; Prasad et al., 2015). Therefore, we investigated whether the increase in WIP1 expression during *in vitro* postovulatory aging occurred in maternally aged oocytes. To this end, we collected MII oocytes from young (8 weeks) and old (12 months) mice (hereafter referred to as *maternally aged oocytes*) and determined their WIP1 expression. Consistent with the increased WIP1 expression found during *in vitro* aging of oocytes, WIP1 levels were upregulated in the maternally aged oocytes (Figure 6A). Moreover, compared with the young oocytes, the increase in *γ*-H2AX levels after etoposide treatment was marginal in maternally aged oocytes. However, *γ*-H2AX levels increased after 1 h of recovery, and that increase was more pronounced when WIP1 was inhibited during recovery, similar to the results obtained from *in vitro* aged oocytes (Figure 6B). In contrast to the *γ*-H2AX levels, the increased TUNEL signals after etoposide treatment decreased after 1 h of recovery, and that decrease was more dramatic when WIP1 was inhibited during recovery (Figure 6C). Furthermore, oocyte fragmentation was reduced by WIP1 inhibition, implying that WIP1 inhibition improved oocyte quality (Supplementary Figure S8). Our results thus demonstrate that the increased WIP1 expression that occurs with maternal aging suppresses the capacity of oocytes to respond to and repair DNA damage, and that WIP1 inhibition also enhances DNA repair capacity in maternally aged oocytes (Figure 6D).

## Discussion

Rapid and concerted signaling in response to DNA damage is of paramount importance in processing DNA damage and preventing adverse consequences to cellular function and survival. However, the ability of cells to process such signaling declines with age. Despite significant efforts, no one yet understands why DNA damage accumulates with age or what molecular mechanism is responsible for the age-associated decline in DNA repair capacity. In this study, we found that the decline in DNA repair capacity in aged oocytes is caused by an increase in the WIP1 level that occurs during aging. Therefore, WIP1 inhibition could reverse the decrease in DNA repair capacity in aged oocytes, which would improve oocyte quality and developmental competence.

The formation of *γ*-H2AX foci is the one of the earliest events in the cellular response to DNA DSBs (Rogakou et al., 1998). Because of its early appearance and essential role in the DSB response, *γ*-H2AX is a sensitive biomarker for DNA DSBs. However, we found that in aged oocytes, the *γ*-H2AX signal did not increase immediately in response to DNA DSBs. Instead, the *γ*-H2AX signal increased slowly during recovery from DNA damage. Furthermore, despite the slow increase in *γ*-H2AX signaling, the TUNEL signal did not change significantly, suggesting that aged oocytes have little capacity to repair DNA damage and that *γ*-H2AX signaling does not reflect the level of DNA damage in aged oocytes. Because WIP1 negatively affects DNA damage repair in immature oocytes (Leem et al., 2018), we focused on WIP1 to discover why aged oocytes have reduced capacity to respond to DNA damage. Surprisingly, we found that the WIP1 level increased significantly as oocytes aged, implying a possible association between WIP1 and the decline in DNA damage response in aged oocytes. Indeed, we observed that a forced increase in WIP1 expression in oocytes decreased their capacity to respond to DNA damage. Therefore, our results suggest that increased WIP1 is closely related to the decline in DNA damage response in aged oocytes.

After DSB formation, the MRN complex binds to the DSB site and targets ATM to initiate *γ*-H2AX signaling, which in turn expands the signal to adjacent chromatins by using MDC1 as a mediator (Stucki et al., 2005; Polo and Jackson, 2011; Panier and Boulton, 2014). In this study, we found that in aged oocytes, MDC1 did not immediately increase in response to DNA damage, but increased slowly during recovery, which is consistent with the delayed increase in *γ*-H2AX signaling during recovery in aged oocytes. Moreover, the recruitment and activation of ATM in the chromatin and the subsequent spread across chromatins were severely impaired in aged oocytes. Thus, our results suggest that the ability to propagate *γ*-H2AX signaling to neighboring chromatins in response to DNA damage is significantly impaired in aged oocytes. Importantly, the delayed increase in *γ*-H2AX and MDC1 signaling during recovery also appeared in WIP1-overexpressing oocytes, suggesting that the age-related impaired amplification of *γ*-H2AX signaling is closely associated with increased WIP1 levels. Given that ATM is a substrate of WIP1 (Shreeram et al., 2006), it is likely that increased WIP1 in aged oocytes directly blocks ATM activation, which hampers the amplification of *γ*-H2AX signaling. Therefore, WIP1 inhibition could reactivate ATM and thus *γ*-H2AX signaling in aged oocytes. Indeed, the effect of WIP1 inhibition was abolished by ATM inhibition, supporting the direct connection between WIP1 and ATM. However, we cannot exclude the possibility that WIP1 indirectly regulates the chromatin structure in a way that disturbs the formation of *γ*-H2AX foci and subsequent signal amplification. Consistent with that notion, aging is accompanied by changes in the chromatin structure, which are also likely to influence *γ*-H2AX signaling (Feser and Tyler, 2011). Moreover, there is evidence that DSB sensing is triggered by an alteration in the chromatin structure that promotes the recruitment of repair proteins by relaxing the chromatin fiber (Murr et al., 2006; Downs et al., 2007). Further studies are thus required to clarify the action of WIP1 in the DNA damage response in aged oocytes.

A series of morphological and cellular changes that occurs during oocyte aging could affect not only fertilization and early embryo development, but also the later life of the offspring (Miao et al., 2009). Therefore, efforts have been made to delay oocyte aging, which is particularly important to increase the rates of successful outcomes in assisted reproductive technology (ART) procedures. In this study, we found that WIP1 inhibition during *in vitro* culture prevented aging-associated deterioration of oocyte quality and decreased the accumulation of DNA damage, which in turn improved fertilization and early embryo development. Therefore, we propose that suppressing WIP1 is a potential way to delay aging and mitigate age-associated declines in DNA damage response and repair, which ultimately improves oocyte quality and competence. Given that the success of ART is greatly affected by oocyte quality, our results provide interesting insights for clinical settings, such as the possibility of supplementing culture media with a WIP1 inhibitor during ART procedures.

In addition to *in vitro* aging, oocytes experience quality decline in the ovary during maternal aging (Cimadomo et al., 2018). Our data demonstrate that WIP1 expression also increased in maternally aged oocytes, and WIP1 inhibition reversed the decline in DNA damage response and repair in oocytes from aged mice. In contrast to our data, previous studies reported that WIP1 expression decreased significantly in aged mice and that WIP1-deficient mice showed a premature aging phenotype (Choi et al., 2002; Demidov et al., 2007; Wong et al., 2009). Considering that oocytes remain arrested for a long period without proliferation in the ovary, whereas somatic cells are frequently reproduced and replaced through continuous cell division, it is possible to speculate that the increase we found in WIP1 expression during aging is confined to non-dividing cells with long lifespans, such as oocytes. However, the reason that WIP1 increases in aged oocytes needs to be determined through future studies. Also, the correlation between WIP1 levels and the age-associated decline in DNA repair capacity should be investigated in various cells and tissues.

In conclusion, our results reveal that increased WIP1 expression with aging is responsible for an age-associated decline in DNA damage response and repair in oocytes, and they also demonstrate that WIP1 inhibition is sufficient to increase DNA repair capacity in aged oocytes, which in turn improves oocyte quality. Our data advance our understanding of the age-associated decline in DNA damage response and repair in oocytes and provide ways to establish methods to reverse or delay oocyte aging.

## Data Availability

The original contributions presented in the study are included in the article/Supplementary Material, further inquiries can be directed to the corresponding author.
