# Supplementary material for: Increased WIP1 Expression With Aging Suppresses the Capacity of Oocytes to Respond to and Repair DNA Damage
Source: Front Cell Dev Biol. 2021 Dec 24;9:810928. doi: 10.3389/fcell.2021.810928 (PMC8740286; doi:10.3389/fcell.2021.810928)
Supplement: Supplementary file 1 [file DataSheet1.PDF]

Fig. S1

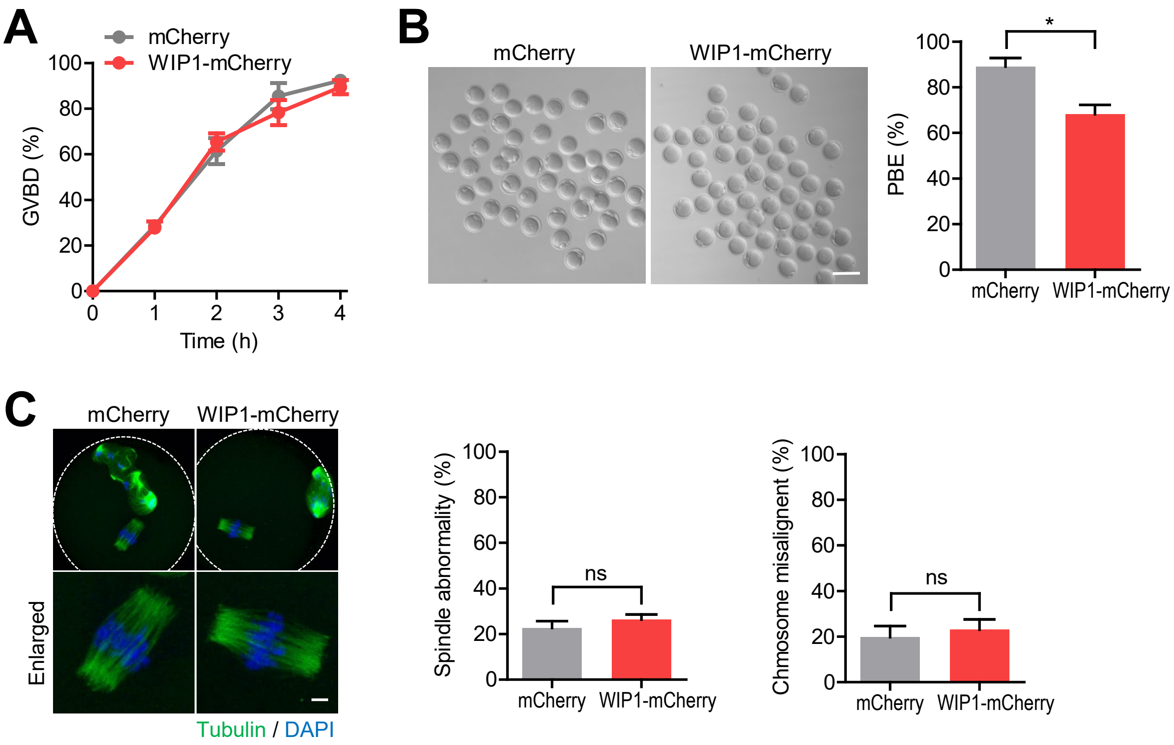

Fig. S1. Overexpression of WIP1 in mouse oocytes. (A–C) GV oocytes were injected with mRNA encoding mCherry or WIP1-mCherry and matured to the MII stage for 16 h. (A, B) The rate of GVBD and polar body extrusion (PBE) was scored, and representative images are shown. (C) After *in vitro* maturation, MII oocytes were subjected to immunofluorescence staining with α-tubulin and DAPI. The percentage of oocytes with spindle abnormalities or chromosome misalignment was quantified, and representative images are shown. Scale bar, 10 μm. *$P < 0.05$; ns, not significant.

Fig. S2

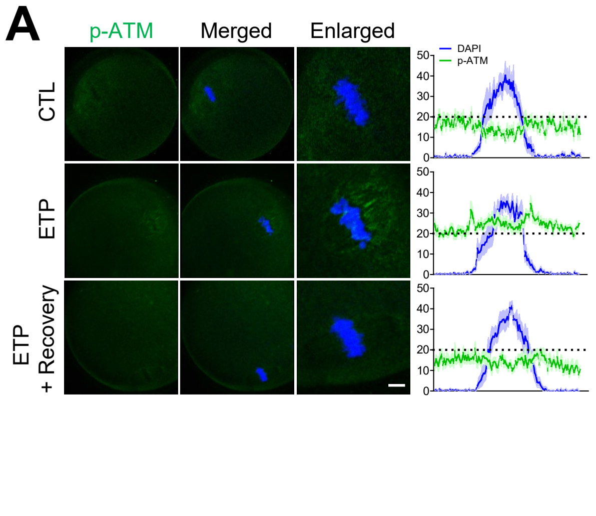

Fig. S2. Activation of ATM signaling in fresh and aged oocytes in response to DNA damage. After 15 min of exposure to etoposide (ETP) or DMSO as a control (CTL), fresh oocytes (A) and oocytes aged *in vitro* for 24 h (B) were cultured in ETP-free medium for up to 1 h for recovery. They were then immunostained with the p-ATM antibody and DAPI. Line scan analysis of p-ATM signals are shown with representative images from three independent experiments. Scale bar, 10 μm. The threshold levels of p-ATM in line scan plots are indicated by the dotted lines.

Fig. S3

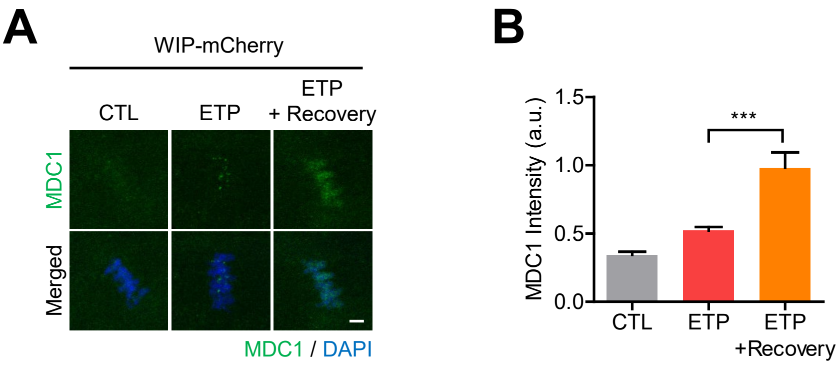

Fig. S3. Delayed increase in MDC1 levels during recovery from DNA damage after WIP1 overexpression. (A) *In vitro*–matured MII oocytes overexpressing WIP1 were exposed to ETP for 15 min and allowed to recover from the DNA damage for 1 h in ETP-free medium. They were then immunostained with the MDC1 antibody and DAPI. Scale bar, 10 μm. (B) The intensities of the MDC1 signals normalized to the mean DAPI intensity are shown. Data were analyzed by one-way ANOVA followed by Tukey's post hoc test and are expressed as the mean ± SEM of three independent experiments. ***P < 0.0001.

Fig. S4

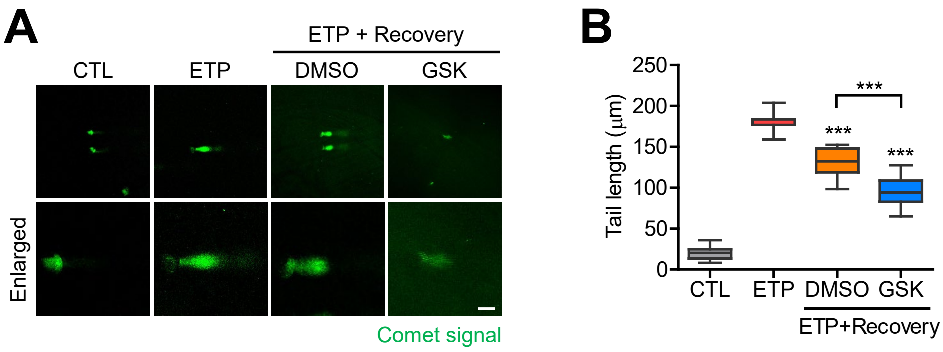

Fig. S4. WIP1 inhibition enhances the repair of damaged DNA. (A) MII oocytes were aged *in vitro* for 24 h and then exposed to ETP for 15 min. After being washed, the oocytes were cultured in ETP-free medium with GSK2830371 (GSK) or DMSO for 1 h of recovery. They were then subjected to the comet assay. Scale bar, 10 μm. (B) The tail length of the comet was scored and is expressed as the mean ± SEM of three independent experiments. Data were analyzed by one-way ANOVA followed by Tukey's post hoc test. ***P < 0.0001.

Fig. S5

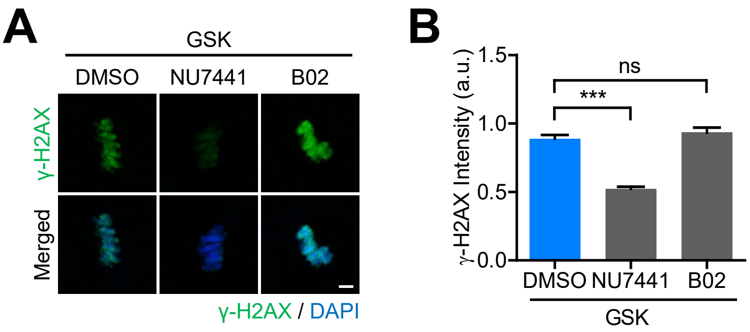

Fig. S5. Increase in γ-H2AX signals induced by WIP1 inhibition in aged oocytes is abolished by inhibiting the NHEJ repair pathway. (A) After 15 min of ETP exposure, MII oocytes aged *in vitro* for 24 h were cultured in ETP-free medium with GSK2830371 (GSK) for 1 h of recovery. During recovery, the oocytes were treated with NU7441 or B02 to block the NHEJ or HR pathway, respectively. The oocytes were then immunostained with the γ-H2AX antibody and DAPI. Scale bar, 10 μm. (B) The intensities of the γ-H2AX signals are shown normalized to the mean DAPI intensity. Data were analyzed by one-way ANOVA followed by Tukey's post hoc test and are expressed as the mean ± SEM of three independent experiments. ***$P < 0.0001$; ns, not significant.

Fig. S6

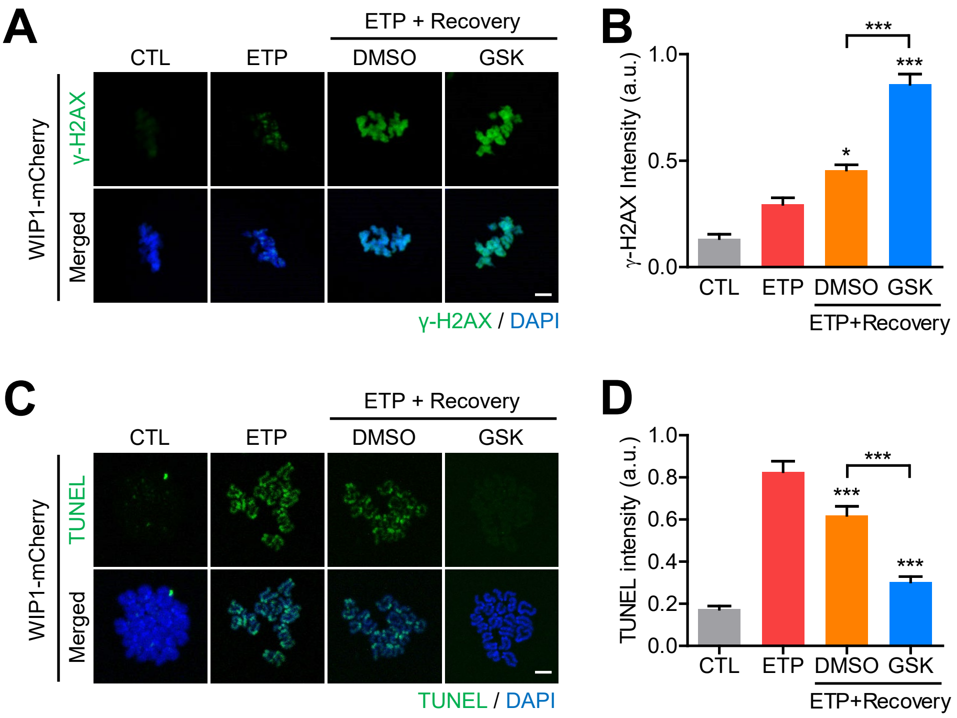

Fig. S6. WIP1 overexpression impairs γ-H2AX signaling and subsequent DNA damage repair. (A, C) *In vitro*–matured MII oocytes overexpressing WIP1 were exposed to ETP for 15 min and allowed to recover from the DNA damage for 1 h in ETP-free medium containing GSK2830371 (GSK) or DMSO. The oocytes were then subjected to γ-H2AX staining or the TUNEL assay. Scale bar, 10 μm. (B, D) The intensities of the γ-H2AX and TUNEL signals are shown normalized to the mean DAPI intensity. Data were analyzed by one-way ANOVA followed by Tukey's post hoc test and are expressed as the mean ± SEM of three independent experiments. $^*P < 0.05$; $^{***}P < 0.0001$.

Fig. S7

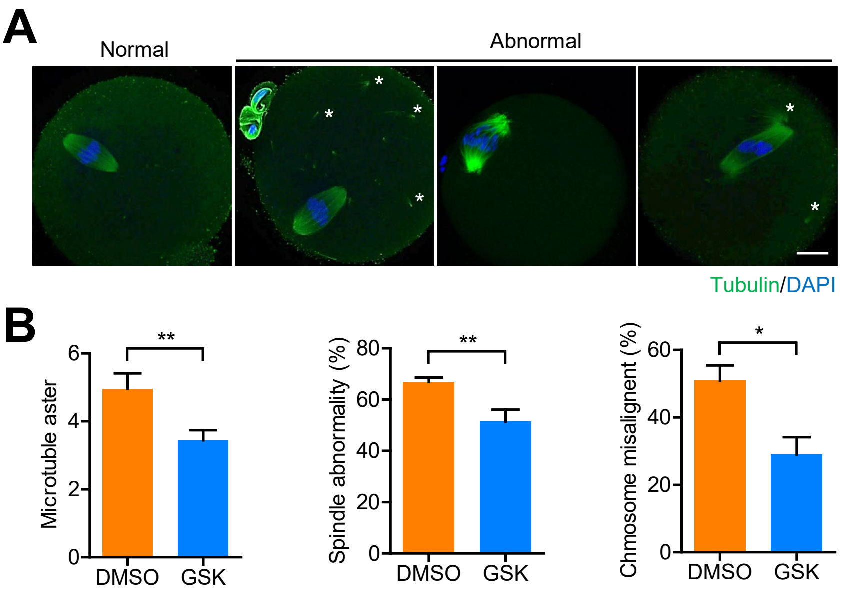

Fig. S7. WIP1 inhibition decreases aging-associated decline in oocyte quality. (A) MII oocytes were cultured *in vitro* for 24 h with GSK2830371 (GSK) or DMSO. They were then immunostained with α-tubulin and DAPI. Scale bar, 20 μm. Microtubule aster is marked by an asterisk. (B) The number of microtubule asters and the percentage of oocytes with spindle abnormalities or chromosome misalignment were scored and are expressed as the mean ± SEM of three independent experiments. *P < 0.05; **P < 0.001.

Fig. S8

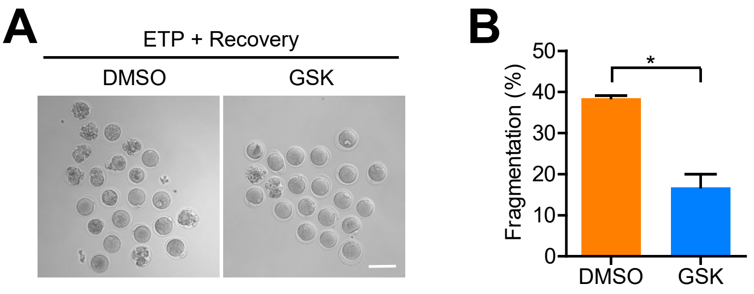

Fig. S8. WIP1 inhibition decreases the fragmentation of maternally aged oocytes during recovery from DNA damage. (A, B) MII oocytes from 12-month-old mice were treated with ETP for 15 min and allowed to recover from DNA damage for 1 h in ETP-free medium with GSK2830371 (GSK) or DMSO. The fragmentation of oocytes was scored, and representative images are shown. Scale bar, 100 μm. *$P < 0.05$.
